# A Cycle of Social Violence: a novel theoretical framework for explaining how structural, slow, and symbolic violence interact to produce and maintain health inequalities in England

**DOI:** 10.1016/j.socscimed.2025.118438

**Published:** 2025-07-19

**Authors:** T.J. Price, V.J. McGowan

**Affiliations:** Population Health Sciences Institute, Faculty of Medical Sciences, Newcastle University, Newcastle upon Tyne, NE1 4LP, UK

## Abstract

Health inequalities are a form of violence, produced and sustained by political, economic, and social structures that systematically disadvantage certain communities. Drawing on qualitative data from 194 participants in six English towns, this study develops the Cycle of Social Violence, a novel theoretical framework that conceptualises how structural, slow, and symbolic violence interact to create and perpetuate health inequalities. Participants’ narratives illustrate how structural violence, driven by neoliberal economic policies creates the material conditions for poor health. These harms unfold over time as slow violence, extending their impacts and making their effects difficult to trace to specific causes. Symbolic violence then legitimises and obscures these injustices, reinforcing narratives that blame individuals rather than structural forces. The interaction of these three forms of violence produces a self-perpetuating cycle that deepens inequalities and erodes resistance to systemic harm. This study highlights how these dynamics manifest in deindustrialised and economically deprived communities, where declining public services, insecure work, and stigma reinforce poor health outcomes. Breaking the cycle of social violence requires policy interventions that incorporate the lived experience of people in affected communities and that go beyond surface-level regeneration to address the root causes of economic and social deprivation.

## 1 Introduction

Health inequalities are a form of violence. Despite sustained interest from both academics and policy makers, little progress has been made in England in closing these inequalities in health (Marmot et al., 2020; Bambra, 2016). In England, men living in the most deprived areas of the country can expect to live 10 years less than their counterparts in the least deprived areas of the country (for women, the life expectancy gap is 8 years) (ONS, 2022). Health inequalities in England are geographic in nature, that is to say, they affect some areas more than others (Bernard et al., 2024). Inequalities in healthy life expectancy persist between regions, cities, and towns, with deindustrialised and coastal communities bearing some of the most dramatic inequalities in health and life expectancy relative to other places in England (Corris et al., 2020; Bambra, 2016). In this paper we draw on three existing theories of violence to extend our understanding about the ways in which health inequalities are produced and maintained, demonstrating a cycle of violence. Drawing on the concepts of structural, slow, and symbolic violence we interrogate how these concepts interact to produce and reinforce inequalities in health. We argue that health inequalities are a form of violence because they are produced by political, economic, and social systems and structures which are amenable to intervention (Bambra et al., 2005; Lynch, 2020).

The concept of structural violence was developed within the field of peace studies; an interdisciplinary field that draws on elements of history, sociology, political science, philosophy, and others (Lawler, 2008; Galtung, 1969). Banerjee et al. (2012) described the concept as “the role that institutions and social practices play in preventing people from meeting their basic needs or realizing their potential” (p.390). Structural violence’s inherent focus on the structural drivers of negative health outcomes is evocative and encourages direct engagement with the fact that health inequalities are a product of social conditions. Thus, the concept has been adopted within health inequalities literature to describe the ways through which government policy leads to ill-health and death (Neely et al., 2020; Zakrison and Britt, 2017; Price et. al., 2024).

Slow violence originates from the field of environmental humanities and is rooted in postcolonial ecological research (Nixon, 2011). Nixon (2011) introduced the term to highlight the gradual, often invisible environmental degradation that disproportionately affects marginalised communities, particularly in the Global South. The concept of structural violence as constructed by Galtung (1969) has an inherent temporal element due to its emphasis on understanding how past injustices influence present living conditions, but it is often implicit and not directly examined (Banerjee et al., 2012; Galtung, 1964, 1969). Slow violence builds on the concept of structural violence by placing greater emphasis on the tendency for violence to go on for an extended period of time without notice (Nixon, 2011; Pain, 2019). While the concept has most frequently been used to describe the impacts of environmental degradation on human health (Davies, 2018, 2022), it has also been applied to studies examining the impacts of neoliberal economic policies on urban environments (Kramer and Remster, 2022; Pain, 2019; Yetiş and Bakırlıoğlu, 2023). The concept of slow violence is significant because it highlights forms of harm that are often overlooked in mainstream discourse due to their protracted and diffuse nature.

Symbolic violence is a concept advanced by sociologist Pierre Bourdieu that explains the ways in which power and dominance are maintained through cultural, rather than physical, force (Bourdieu, 1977). Bourdieu explains that within society, dominant groups impose norms, values, and perceptions upon a subordinate group through language, education, and social institutions, which leads the latter to perceive the social order as just and legitimate (Bourdieu, 1977, 1990). Symbolic violence emphasizes how power operates subtly and pervasively, shaping perceptions, behaviours, and social hierarchies without overt coercion. By focusing on the internalisation of domination, symbolic violence underscores the role of cultural and symbolic systems in sustaining social stratification, often rendering inequality invisible or giving it an appearance of naturalness (Bourdieu, 1990). In public health research, symbolic violence has been used to critique how health promotion campaigns and policies may blame individuals for poor health outcomes, rather than addressing systemic barriers to health (Lupton, 2015; Mikkelsen et al., 2021).

Interactions between structural and symbolic violence are gaining prominence in the literature with growing recognition of how these forms of violence intersect to produce and sustain inequalities or cause harm. While not always framed explicitly in these terms, several studies illustrate how structural systems of power become internalised in ways that inflict harm. Peacock et al. (2014) for example, describe how women’s experiences of inequality are shaped by neoliberal discourses (structural violence) that position individuals as responsible for their own disadvantage (symbolic violence). Similarly, Cleveland et al. (2018) highlight the interaction between structural and symbolic violence in their study of asylum seekers held in Canadian immigration detention centres. They show how structural processes of incarceration and surveillance are reinforced by narratives that dehumanise asylum seekers, legitimising their exclusion and exacerbating psychological distress (Cleveland et al., 2018). Indeed, as there are also examples that demonstrate the interaction of slow and structural violence (Davies, 2022; Pain, 2019; Yetiş and Bakırlıoğlu, 2023). However, to our knowledge, there has been no examination of the ways in which these three forms of violence interact to produce and maintain inequalities in health.

To address this gap in the literature, we propose the cycle of social violence, a novel theoretical framework that conceptualises how structural, slow, and symbolic violence interact to produce and sustain health inequalities. Drawing on participants’ accounts across six English towns, the framework demonstrates how political and economic decisions, particularly deindustrialisation, welfare reform, and austerity, constitute forms of structural violence that undermine the material conditions necessary for good health. These harms are not experienced as isolated shocks but unfold gradually, accumulating over time as slow violence, eroding public infrastructure, community cohesion, and individual well-being. In turn, these conditions are legitimised and obscured through symbolic violence, as public and institutional narratives attribute poor health and deprivation to individual failings rather than systemic neglect. We propose that these three forms of violence are not discrete; they operate in concert to produce a self-reinforcing cycle that deepens inequality and diminishes the political and social capacity to resist it. Rather than examining embodied health outcomes, this paper focuses on how residents of communities marked by poor health interpret and explain the persistent inequalities that shape their environments, offering insight into the structural, temporal, and symbolic forces they identify as driving those inequalities.

## 2 Methods

This article draws on qualitative data from a longitudinal mixed methods study examining regional health inequalities between the north and south of England. Two qualitative studies were conducted in six English towns. Data from these studies were combined and reanalysed to examine the ways in which the concepts of structural, slow, and symbolic violence interact to produce and maintain inequalities in health. To ensure anonymity, only the town name will be provided as an identifier in quotes.

Study 1 (led by the 1st author) was a cross-sectional study examining inequalities in deaths due to drug, suicide, and alcohol – so called “deaths of despair” (DoD)– in two towns in North East England (Middlesbrough and South Tyneside) and included 54 participants who lived and/or worked in the towns. Stakeholders (n = 24)) were eligible to participate in this study if their work involved people living in Middlesbrough or South Tyneside and if their work pertained directly or indirectly to drug, self-harm, or alcohol-specific morbidities and mortalities (e.g. mental health and substance abuse treatment, charity service provision, local government, public health practice). Community members (n = 30) were eligible to participate if they lived in one of the recruitment sites. Several purposive sampling techniques were used to recruit stakeholders including advertising in a mailing list and snowball sampling of professional networks. The study was advertised to community members via flyers displayed in spaces frequented by members of the community. The primary researcher in Study 1 (1st author) also recruited participants by visiting community groups (e.g. public living rooms, community drop-ins) and advertising the study. Data were collected through semi-structured interviews and a focus group. Participants were asked how they felt factors such as poverty, government policy, and sociocultural factors contributed to inequalities in rates of DoD in their towns. Data collection was carried out from November 2022 to December 2023. Further details on the recruitment and data collection techniques used in this study, as well as the study’s analytical approach, have been published elsewhere (Price, 2024; Price et al., 2024).

Study 2 (led by the 2nd author) was an ethnography examining the north/south health divide across four coastal towns in England (Hartlepool, Blackpool, Hastings, and Torbay) and included interviews/focus groups with 140 participants who lived and/or worked in the towns. Field work was conducted from April to December 2023 with the 2^nd^ author residing in each town for 4–6 weeks and undertaking ethnographic observations in social and community spaces to aid recruitment. Participants were eligible to take part if they lived and/or worked in any of the four towns and were recruited via email (if publicly available), inperson interactions during ethnographic observations, community events, or via flyers distributed in public spaces, charity and local authority newsletters, and social media. Snowball sampling was also adopted as participants shared study information with colleagues, family and friends. The sample consisted of residents (n = 73), who worked in the voluntary and charity sector (n = 34), in the public or private sector (n = 19), voluntary and charity sector non-residents (n = 4) and public or private sector non-residents (n = 10). Participants were asked about their experiences and perceptions of health inequalities in their towns and explored themes such as area perceptions, migration, employment, housing, education, health, community cohesion, and economic deprivation. Further details on recruitment, data collection, and analysis for Study 2 has been reported elsewhere (McGowan, 2025).

### 2.1 Reflexivity and researcher positionality

In conducting the studies upon which this article is based, we were aware of the power asymmetries between researchers and participants, particularly given that we were conducting research in communities experiencing long-term structural disadvantage. To mitigate these imbalances, we adopted a relational and reflexive approach throughout fieldwork. In both studies, interviews were designed to be conversational, with participants encouraged to shape the direction of discussion and reflect on what mattered most to them. Interviews were conducted in familiar community settings and conducted after we had formed relationships in those areas, which helped build trust and reduce hierarchical distance. We also emphasised voluntary participation, provided clear information about the research aims, and remained transparent about how participants’ data would be used. Throughout data collection and analysis, the authors held regular reflexive discussions to consider how our own positionalities may have shaped participants’ responses and our interpretations. While this paper primarily focuses on shared place-based experiences of structural violence, we recognise that these are further shaped by intersecting identities such as gender, race, age, and disability. While intersectionality was not the primary analytic frame in this paper, we view it as an essential lens for future research building on this work.

## 3 Results

Across the six field sites, participants’ narratives highlighted how health inequalities stemmed from structural violence. These accounts also revealed how such inequalities were internalised as symbolic violence, with structural disadvantage reframed as inevitable. This was further compounded by slow violence, as prolonged economic decline eroded resources, hope, and wellbeing. While some focused on specific forms of violence, others described their interaction, structural deprivation fostering symbolic violence, which in turn deepened slow violence by weakening resistance and embedding inequalities. While there were minor variations in how, and to what extent, each form of violence manifested across the towns, participants consistently conveyed a coherent and compelling picture of how structural, slow, and symbolic violence shaped the inequalities present in their communities.

### 3.1 Structural violence

Participants in both studies consistently linked deprivation, poor health, and inequalities they experienced in their towns to structural factors. Their narratives conveyed a shared understanding that the ultimate causes of these issues were not due to individual failings (although some did express individualised views which are discussed below) but in political decisions, economic systems, and the withdrawal of state support. These structural explanations reveal how persistent neglect of their towns, compounded by economic restructuring and austerity, has inflicted harm on their communities, a form of structural violence. Participants rarely viewed these health and social inequalities as inevitable or natural but articulated how political action and inaction had deliberately produced and sustained disadvantage. One participant in Hastings felt this was an active decision by those in power:

“I just feel I think there is no desire to reduce inequality. I think there’s an active- the actions they are taking suggest to me that there’s an active desire to increase inequality and I think they’re scooping out the middle classes now, personally … if you look into it all, it’s all about that’s how society works in this country, isn’t it? We keep this gap between rich and poor” (Hastings).

Deindustrialisation was a prominent theme across most of the participants’ narratives, economic restructuring was seen as a key driver of deprivation and poor health. While the affected industries varied, participants consistently described how their towns had been left behind as the national economy shifted towards finance and services. These changes were not seen as inevitable, but as a result of political decisions that dismantled local economies without providing meaningful alternatives. The long-term effects of deindustrialisation, amplified by austerity, were understood to have profoundly shaped the health and wellbeing of their communities.

“I think at the heart of it, it’s to do with jobs and the economy. I think that politicians of all stripes have never- In the last 30, 40 years, I think the economy in places like Hartlepool has changed so radically. Places like Hartlepool used to be the engine room of the British economy, which meant that there were generational jobs there in the industries that surrounded. And obviously from the 1980s onwards, a lot of that was stripped away with no real thought of how to replace it. And subsequent attempts, so they say about lots of different politicians, including the current crop, is this idea of, “We’ll give you handouts, we’ll give you capital grants. We’ll make your railway station look nice, we’ll make that building over there look nice.” And that’s not going to solve the problem” (Hartlepool).

Participants in Hartlepool and Middlesbrough articulated this austerity amplification as creating a cycle of disinvestment and systemic neglect. In Middlesbrough, one participant reflected on how austerity had resulted in service funding becoming more fragmented and short term, making it increasingly difficult to support people. This shift reduced the capacity of public services to meet the complex needs of the population, intensifying the challenges faced by those living in disadvantaged circumstances.

“Funding is still quite, probably as a result of austerity, it’s even more siloed than it was before. It’s quite difficult to look at the whole person, or the whole family, across a range of services and support. Less people employed to consider policy from a broader perspective. It’s short-term too. We might get a grant for 12 months, or 18. We have to be hyper-focused, more focused on operational efficiency which is the opposite of building connections with other services.” (Middlesbrough).

Similarly, in Hartlepool, participants describe the town’s decline as a result of both deindustrialisation and austerity. Once a thriving industrial hub, Hartlepool has seen its key industries disappear, with one participant pointing to Thatcher-era policies of privatisation and industrial closures as the root cause. The town has struggled with stagnation for decades, but the impact of austerity has compounded this decline. Austerity measures were described as ‘horrendous’ and have deepened the sense of abandonment, leaving the town in a cycle of neglect and disinvestment.

“How has it gone from being the third most popular port in the world to this, to nothing? We were an industrial town, all that industry went, or most of it has gone … nothing has changed for 30 years, the last 13 years, the austerity measures that have been put in have just been horrendous but this building on the back of decades of neglect and mismanagement … Margaret Thatcher, she closed all the stuff, all the industry, she privatised it all and got rid of it” (Hartlepool).

Participants across all towns made reference to various regeneration schemes intended to stimulate the local economy, and reverse the decline caused by deindustrialisation. However, there was a widespread sense that these schemes focused primarily on improving the public realm, enhancing retail options, or beautifying areas to attract visitors, while the root causes of the decline were neglected. Despite these surface-level improvements, participants emphasised that such efforts had not restored jobs lost during deindustrialisation or improved quality of life for residents. One participant in Hastings argued that these types of interventions are insufficient to tackle the root causes of inequalities in their town:

“Well, you need big solutions for big problems. It is just tinkering around the edges, the way various governments have done with regeneration pots of funding which don’t really regenerate anything much at all, really. They might tart things up and get people- yes, their properties look slightly better for a few years, and then we are back to the same old, same old, unfortunately” (Hastings).

As a result, these regeneration initiatives often felt like a form of managed decline, where cosmetic changes did little to address deeper economic and social challenges facing the communities. One participant from South Tyneside reflected on the previous government’s ‘Levelling Up’ scheme, asserting that it had done little to rebalance the regional divides, leaving the town’s core issues unresolved, namely a lack of good employment opportunities.

“They talk about levelling up, but … I mean I could talk about that till the cows come home, but it hasn’t worked. There just isn’t a lot of opportunities here. There is no engineering jobs or anything, you’ve got to go down South to do that sort of work and why can’t they be up here? Why can’t we have the same opportunities down South has?” (South Tyneside).

There were common themes of disillusionment with the political and economic systems that participants believed were designed to maintain the status quo, further embedding inequalities. In Middlesbrough, one participant suggested that the system is resistant to change, reflecting a widespread perception that the system is fundamentally flawed with those in power benefiting from maintaining inequality. The comparison to historical periods suggests that while the reasons for poverty and deprivation may have evolved, the underlying structures of exploitation and neglect remain largely unchanged.

“I don’t think it will change the way the system is. The way the government is. They don’t even bother, people don’t, they think everyone is lining their pocket and poverty will always be there. It’s not any better than the 18^th^, 19^th^, 20^th^ centuries, just the reasons are different” (Middlesbrough).

Similarly, in Torbay, a participant described the town’s trajectory as part of a broader national decline where incremental changes, rather than bold decisions, leave communities stuck on a path of worsening conditions. They recognise that the system lacks the will or the ability to address deep-rooted issues. This sense of inevitability, that things will not improve without substantial intervention, further supports the idea that the system not only resists change but actively maintains the conditions of structural violence.

“I think unless there are bolder decisions taken then, you know, there might be some minor improvements but otherwise we’re still on a course of decline which is national, that’s not unique to Torbay, but we’re going to feel it probably that much more” (Torbay).

### 3.2 Slow violence

Slow violence was embedded in narratives from the six towns in the way participants connected social and economic decline with long-term health outcomes. The erosion of stable jobs, particularly during the 1980s under Margaret Thatcher’s government, has left communities struggling with the loss of economic opportunities and a sense of abandonment. As major industries closed or reduced their workforces, large-scale employment vanished, and the few new technology jobs introduced were insufficient to replace the lost work. This decline has contributed to a sense of hopelessness with many participants describing how the closure of public spaces, the shuttering of local businesses, and the rise in issues such as alcohol dependency have caused declines in mental and physical wellbeing. The ongoing deterioration fosters frustration, anger, and a feeling of being left behind, with people noting that the slow collapse of their communities continues to have a lasting impact on their lives and the future of their towns. For example, a participant in Hartlepool connects longer term poor health with processes of deindustrialisation which began decades ago, indicating the slow violence being inflicted on this community.

“Post-industrial, which is pretty well most of the north east, the whole of the town. You know, no new businesses coming in to take over, or very few new businesses coming to take over. I think that’s where you see you’ve had most of the industry, the shipyards, historically. That obviously drives some poor health, as well, in the longer term” (Hartlepool).

This slow decline of once-thriving communities has occurred over decades, with the structurally violent process of deindustrialisation being extended through time and space, leading to the erosion of wider industries such as retail and leisure. One participant in Middlesbrough quite simply states, ‘*the industry died … the towns went right down, even now from then’* demonstrating the temporal stretch of deindustrialisation.

“Oh, there was [jobs available] in 70s. The shipyards, ICI, but that’s all gone. Same with mining out of Middlesbrough. It’s all gone, so the communities are dying. There are some areas a lot worse than Middlesbrough but I cannae see the next five to ten years getting better, I just cannae envisualise [sic] it. It was hard and we never really recovered from that. The industry died. … The towns went right down. Even now, from then.” (Middlesbrough).

In Blackpool, one participant describes their surprise at how much the town had declined after returning to live in the area after two decades living away. They describe a once bustling town, with streets filled with shops and markets, which have now given way to derelict spaces. Buildings that were once staples in the community, like the former high-street shop Woolworths, are now abandoned and forgotten, standing empty and decaying as time passes. This slow violence, the ongoing neglect of these towns relates not just to the loss of industry but also the gradual disappearance of social life from these once bustling streets.

“As soon as I got back I couldn’t believe how much worse it had got, I thought it had got quite bad when I’d left but then when I come back I couldn’t believe how much it had changed for the worse, I’d left 20 years ago and nobody had done anything since but they’d not maintained it either so it had just got worse. So up on Lytham Road when I was a child there was just shops, markets, all the way up to town, and then moving back there’s nothing, it’s all shut down all empty and its like, did you notice that building on the corner that’s sort of derelict so that used to be Woolworths and then it was a market after that and then that’s just been left, there’s a lot of shops empty shops that are left” (Blackpool).

Another participant from Middlesbrough articulates how this slow violence personally ‘*plays on me’* indicating the mental harm being inflicted by the social decay of their town. There is a sense of frustration from seeing their town fall into disrepair and social spaces such as community centres slowly retreating through reduced opening hours. The feeling of being abandoned by both local government and society at large contributes to a deep sense of hopelessness, as shops close and vacant buildings exacerbate the decay of both the physical space and community spirit. The slow violence of economic decline has become a constant presence in these towns, undermining their economies as well as their social and cultural vitality.

“It plays on me, my community is closing down all around me. You go pass another thing that was there one day and now its closed. Shop here, shop there, all closed. Walking in the Grove Hill Hub on a Thursday and you’re told you cant come in. You know? They say its closed to the public now on Thursday. They still do classes and stuff then, so its open but its closed. Can’t go in, can’t use the toilet, can’t do anything. It basically all gets to you bit by bit. It gets to me. Its like, it makes me angry and irritated at the council and the government and why this is happening. Its having an effect on the public, on me. It contributes to that feeling of hopelessness” (Middlesbrough).

In Southern towns like Torbay and Hastings the decline is equally as stark. Once a popular tourist destination known for its lively seafront and vibrant atmosphere, Torbay now faces the slow erosion of these industries. Areas that were once bustling with activity filled with shops and entertainment are now deemed unsafe and neglected, with town centres hollowed out as shops steadily close. This participant expresses concern that this slow decline will continue to get worse.

“It’s changed vastly since I’ve been here. It used to be a lovely place. It was gorgeous down the seafront and we had bands on and we had loads of entertainment and crikey knows what and, now, it’s going downhill fast. It needs something definitely done. Castle Circus is a mess. It’s not safe. You used to be able to walk anywhere and do anything or whatnot. If something’s not done quick … There are lots of shops empty. If something’s not done soon, sorted out, it will probably get worse” (Torbay).

### 3.3 Symbolic violence

Participants frequently described how the inequalities in their towns, that were caused by structural violence, were rendered invisible or reframed in ways that obscured their root causes. This process, whereby the realities of poverty, social exclusion, and economic injustice are normalised, misrepresented, or blamed on those experiencing them illustrates symbolic violence. Rather than recognising the broader systematic factors that create deprivation, public narratives often deflect attention onto individuals reinforcing stigmatisation and division. Participants highlighted how media portrayals, political rhetoric, and everyday discourse contributed to this process, shaping public perceptions of poverty and disadvantage in ways that maintain existing power structures. For example, a participant from Blackpool critiques the selective nature of public scrutiny, noting that while working-class communities are sensationalised and vilified in programmes like *Benefits Street*, the financial misconduct of the wealthy, such as tax evasion, is rarely given the same moral weight. By highlighting this inequality, they expose how symbolic violence functions to direct public anger towards the most visible forms of poverty while deflecting attention from the systemic exploitation that perpetuates inequality.

“It’s like ‘Benefits Street’. They put a programme on telly about people and it’s like, “Whoa.” It’s getting people’s blood boiling. What they should do is ‘Tax Evasion Street’ where you’ve got Jacob Rees-Mogg sending his money out of the country, not contributing towards the NHS or the state. You shouldn’t be allowed to represent your country if you don’t follow the rules” (Blackpool).

Likewise, a participant from Middlesbrough describes how blame is routinely displaced onto marginalised groups, such as refugees and asylum seekers, reinforcing division rather than addressing underlying social problems such as poverty. By framing deprivation as the fault of ‘outsiders’ rather than systemic failings, symbolic violence sustains a cycle of exclusion and marginalisation.

“Then you get people who just want to justify what is going on. They’ll blame refugees and asylum seekers. Say it’s their fault. So of course, they feel excluded. But at the end of the day, they’re a massive part of our community now. It’s easy, isn’t it? No one has to take responsibility for themselves and what’s going on, just blame somebody else” (Middlesbrough).

Participants describe how the widespread visibility of drug and alcohol use in their towns has become increasingly normalised, particularly among younger generations. This normalisation can be understood as a form of symbolic violence, where the pervasive harms of poverty and deprivation are internalised as routine features of everyday life. The erosion of social norms around substance use is not simply a shift in attitudes, but a reflection of how structural disadvantage creates environments where harmful behaviours are both more visible and tolerated. What might once have been viewed as deviant or transgressive becomes commonplace in contexts where few other opportunities for social participation or escapism are available.

“People just don’t think twice about smoking [cannabis] away in front of you, so you’re at risk of taking in what they’re smoking. You know, second hand. There are, a lot of them are kids … They will just walk right down the main street, smoking, without a care. It’s a definite change in what people think is okay.” (South Tyneside).

This symbolic violence operates across social gradients, shaping the ways different forms of substance use are perceived and judged. In more affluent areas, drug use is often rendered invisible or excused as recreational, while in poorer communities it is hyper-visible and pathologized. This narrative from Blackpool exposes how patterns of drug use cut across social class but points to the ways in which symbolic violence deflects blame away from more privileged users while reinforcing stigmatising narratives in deprived areas. The unequal moral framing of substance use reflects how structural inequalities are both reproduced and concealed, embedding the conditions that make drug use a routine part of life in some communities while obscuring the wider forces that sustain these conditions.

“All the negative rhetoric you want to pile in, all the individual blame, all the right realism you want to project onto us that it’s all our fault because, like, “Oh, you drink on Central Drive,” you know, it’s just culture as well. “You drink on Central Drive, you’re a scumbag, but if you sniff coke in the bars of London, you’re a winner.” (Blackpool).

The participant below from Middlesbrough demonstrates symbolic violence in the way assumptions and judgements about poor parenting are projected onto people living in disadvantaged circumstances. These judgements are rooted in internalised moral and cultural values about what constitutes ‘acceptable’ behaviour, particularly regarding parenting and work ethic. The participant in Middlesbrough attributes children’s cannabis use to ‘accepted’ behaviour in the home, implicitly blaming parents for ‘allowing’ their children to partake in substance use. This is symbolic violence as it frames these behaviours as a moral failing of parents without considering the broader socio-economic context, such as poverty, lack of opportunities, and systemic inequalities, that contribute to this situation.

“There are a lot of children smoking cannabis in this town. It’s probably because it’s accepted at home. Their parents are smoking at home, and they give it to their kids. It’s so widely accepted that people have almost forgotten it’s a controlled drug, and rightly so as well because it does affect the brain.” (Middlesbrough).

Similarly, the participant from Torbay reflects symbolic violence in how working-class families are portrayed as having the ‘wrong attitude’ towards work and parenting. That the participant’ calls for ‘parenting lessons’ to correct what they perceive as deficient family dynamics demonstrates an implicit judgement that poor parents lack the skills to raise their children ‘properly’. This internalised moral judgement perpetuates symbolic violence by positioning poor parents as inherently flawed, rather than understanding the effect of broader structural factors. In doing so, it obfuscates the structural causes of poverty, if one accepts that poverty is the fault of the impoverished, one can ignore the systemic factors that shape their lives.

“The big problem I think is the poorer people who don’t have the right attitude to wanting to work, or anything like that. They don’t want to work. A lot of the children at schools, if they have parents who don’t work, they also only aspire to being on benefits when they grow up. What I do think is necessary, and I’ve brought it up at a couple of council meetings, but basically, the last time I spoke about it, the Mayor of [redacted] shouted me down. I said, “What we need here is, we need parenting lessons.” Because when young parents have a baby, we need parenting lessons to stop the rot and teach them how to bring up their children properly, how to deal with relationships, family relationships, relationships with neighbours” (Torbay)

Echoing Peacock et al. (2014), participants described how structural inequalities become symbolically embedded through processes of self-blame and stigma. In Hartlepool, one participant highlighted how the failures of structural systems – such as the welfare system and housing services – are often internalised as personal shortcomings rather than the result of wide socioeconomic forces. This narrative reflects how symbolic violence operates to obscure the structural causes of poverty, redirecting blame onto individuals.

“they feel like they’re the reason that everything is going wrong in their life, they feel like they’re to blame for benefits not going right, for housing not being looked after, and they feel like they’re going to be judged on that” (Hartlepool).

Symbolic violence has permeated institutions due to austerity and reduced local authority budgets. The authority internalises the blame for the lack of resources by framing the situation as a challenge that communities must address on their own. The participant below advocates for communities to ‘take control of their own destinies’, which shifts responsibility for the town’s problems from the authority to the residents. This narrative reinforces neoliberal discourses of individual responsibility and seeks residents to ‘do more for themselves’, thus absolving the authority of its role in addressing systemic problems caused by austerity. In doing so, the authority perpetuates symbolic violence by framing the problem as a matter of individual responsibility, rather than recognising (or acknowledging) structural factors.

“the premise of that is around how do you work with communities to radically take control of their own destinies, kind of stuff. So, there are some opportunities, again, around how do you change a narrative that allows communities to do more for themselves, and almost takes us off the hook as the council for being in charge of everything and having to do everything, as that’s not necessarily the best way forward” (Torbay).

### 3.4 A cycle of social violence

While participants’ narratives often touched upon only one form of violence, relating harm in their community inflicted by structural causes for example, there were examples where their narratives were more complex and nuanced articulating the three forms of violence as interacting. One participant in Blackpool described a sense of alienation that reflects the internalised nature of symbolic violence, where feelings of disconnection from material security are experienced as a personal or collective failure rather than the consequence of structural conditions. They explained that most individuals who are alienated ‘don’t know they’re alienated’ highlighting how inequality becomes normalised as an inevitable reality rather than something that can be ameliorated or eradicated by structural factors. The participant described apathy as a ‘silent killer’ emerging when people lose faith in the possibility of change. Once this faith is eroded, alienation sets in, and the potential for collective action or structural change is diminished, reinforcing the status quo.

“Apathy is the silent killer, it really is. It’s a lack of- the thing is with apathy, it’s the lack of faith to achieve it. Once your faith declines, you’re perfect, because you’re alienated. Once you’re alienated, system change gets removed and things remain the same. It’s creating the alien nation, which is what most people who are alienated don’t know they’re alienated, that’s the thing. It happens, en masse, across our system. Especially for, you know, when I think about material successes as opposed to other sorts of values, I don’t have- the narratives that were once held in society about, “If you work you can get a house, you can have kids, you can have a pension,” that does not- I don’t feel now, if I can be honest with you now, that that doesn’t exist in my reality. It’s certainly not something to aim towards, or something people feel motivated towards. So, it’s not that I’m anti-work, it’s more that the exchange and reward that was once accessible within the system isn’t there. Of course, the boomers will never let you have that. They won’t take that, but like, it’s very true” (Blackpool)

This sense of alienation is bound up with the collapse of the social contract (Wistow, 2022) that once linked hard work to material security. The participant reflects on how aspirations that were once considered social norms, homeownership, secure work, and family life, no longer feel attainable. This is not framed as an outright rejection of work but rather as the recognition that the expected rewards of labour have been withdrawn, making traditional routes to security feel futile. The participant’s frustration at the generational divide, where older generations, ‘boomers’, continue to benefit from these structures, further illustrates how symbolic violence operates by masking structural inequalities as the product of individual failure, deflecting attention away from the broader systems that produce and maintain disadvantage.

The participant’s account also highlights how the erosion of economic security represents a form of slow violence, harm that unfolds gradually over time, often without a singular point of crisis. The steady withdrawal of the social contract has not only stripped away material resources but also depleted people’s capacity to imagine a different future, wearing down collective hope for change. This outcome was particularly evident in one participant in Middlesbrough, who when asked what solutions could be implemented to address the problems present in Middlesbrough, answered that she did not believe the problems could be solved.

“I don’t think it can change. I cannae see it. I think it will decline further and more and more young people will be suffering. Sometimes I think, is there any hope for them? I see lads running round the street for free food. There is young men there, maybe 17, 18 years old and I think, they shouldn’t be stood there. There is older men and women, and I think they shouldn’t be there. I shouldn’t be there, but what will you do? We’ve all got bills at the end of the day, but no work. But it still costs to eat and if you haven’t got the funds, what do they do? I don’t think there is escape for them” (Middlesbrough).

A key function of symbolic violence within the cycle of social violence is its ability to obscure the root causes of inequality by redirecting public attention toward moralized or narratives. Crucially, this does not occur in isolation; rather, it is through the interaction of symbolic and structural violence that these harms are sustained. Symbolic violence reinforces structural violence by shaping public perceptions in ways that justify or deflect attention from systemic harm. Rather than addressing the structural violence of neoliberal economic policies, such as austerity, privatisation, and the systematic underfunding of public services, symbolic violence shifts the focus onto scapegoated groups, reinforcing division and preventing collective resistance. As one participant in South Tyneside observed, political rhetoric often emphasizes issues like immigration to divert attention away from the accumulation of wealth and power among elites:

“At the same time, the government wants us worried about small boats, not worried about Michelle Mone’s [a member of the House of Lords] yacht. This is a government that likes big boats, doesn’t like small boats. If we focus on the small boats, then we don’t notice them and their mates buying yachts. That’s really where the failure comes. They want us distracted from the fact that they couldn’t give a shit about the poor if they tried” (South Tyneside).

This quote highlights how symbolic violence legitimises and perpetuates structural violence by controlling the narratives that shape public perception. By framing social issues in ways that divide communities, such as portraying migrants as a threat while ignoring the economic exploitation and policy failures driving inequality, symbolic violence reinforces existing power structures and prevents scrutiny of the political and economic systems that sustain deprivation. This ability to distort reality and redirect blame is crucial to the reproduction of structural violence, allowing the cycle to continue unchallenged.

## 4 Discussion

**Fig. 1:**
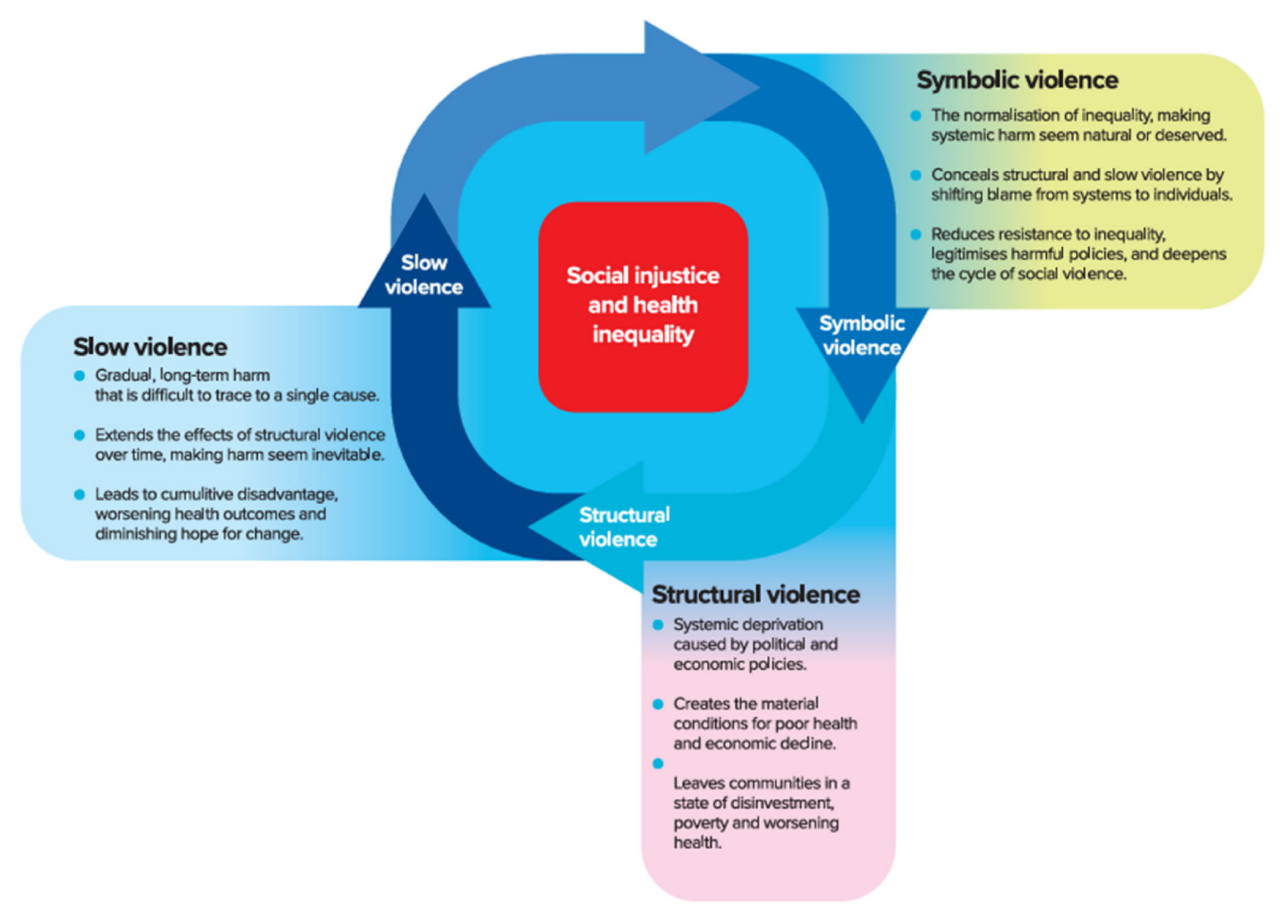
The cycle of social violence.

We propose that structural, slow, and symbolic violence interact to produce the health inequalities present in these towns—this is the *cycle of social violence*, a novel theoretical framework derived from the findings of this study. Fig. 1 illustrates how these forms of violence do not operate in isolation but reinforce one another in a cyclical process that sustains and deepens health inequalities over time. At the foundation of this cycle is structural violence, which participants identified as being driven by neoliberal economic policies, particularly those implemented during the Thatcher administration and through subsequent austerity measures. The temporality of structural violence means its harms unfold gradually, making them difficult for some observers to attribute to a specific cause. Participants further demonstrated how slow violence connects to symbolic violence, as the gradual nature of harm allows for alternative, heavily moralized explanations to take root. In turn, symbolic violence reinforces structural violence, as these explanations are used to absolve social structures of blame and justify further structural harm (e.g., welfare reforms). Through participants’ narratives, we see how structural, slow, and symbolic violence form a self-perpetuating cycle, one that continuously reproduces health inequalities and obscures accountability for the harms inflicted.

This study extends existing health inequalities research by demonstrating how structural, slow, and symbolic violence interact to produce and sustain these inequalities. The role of structural factors, such as economic policy, welfare systems, and labour market conditions, in shaping health inequalities is well established (Bambra, 2016, 2019; Marmot, 2020). Similarly, prior research has highlighted the long-term health consequences of economic disinvestment and austerity (Corris, 2022; Price, 2024; Price et al., 2024), as well as the ways in which stigma and individual blame narratives reinforce health inequalities (Scambler, 2018, 2024). However, this study contributes a novel perspective by conceptualising these processes as interconnected forms of violence, rather than separate or sequential phenomena. By framing health inequalities through the cycle of social violence, this research integrates temporal, structural, and ideological dimensions of inequality into a single explanatory framework. This perspective advances the field by providing a comprehensive account, grounded in participants lived experience, of how disadvantage is maintained, helping to explain why significant health inequalities persist in England despite decades of policy efforts to address them.

The findings of this study highlight not only the material consequences of the cycle of social violence, but also the ways in which certain voices and experiences are marginalised in discussions about health and inequality. This aligns with the concept of epistemic injustice, which describes how some groups are systematically discredited or excluded from shaping knowledge and policy (Chung, 2021; McKinnon, 2016). The interaction of structural, slow, and symbolic violence produces epistemic injustice by silencing the lived experiences of those who experience health inequalities and by framing their suffering through dominant narratives that moralise and individualise the causes of ill health, thereby obscuring the systemic causes of health inequality. This silencing effect reinforces the cycle of social violence, as those most affected by inequalities are denied credibility in academic, public, and policy discourse, limiting their ability to challenge or resist the structures that harm them. Future research must seek to address the epistemic injustices that help to sustain health inequalities, ensuring that the knowledge and perspectives of marginalised communities are recognised and that research outputs can be leveraged to support grassroots efforts to affect change.

This study offers a novel theoretical contribution by conceptualising the cycle of social violence, demonstrating how structural, slow, and symbolic violence interact to produce and sustain health inequalities. This research is grounded in in-depth qualitative data, collected from a wide range of participants in six towns across England, allowing for comprehensive analysis of how these forms of violence manifest in affected communities. There are some inherent limitations to our approach. This study focuses on towns within England, meaning its findings may not capture how these dynamics operate in different national or cultural contexts. While the research highlights the long-term effects of the cycle of social violence, the cross-sectional nature of the data means it cannot fully capture how these processes evolve over time. In developing our framework, we have drawn on data from multiple towns to identify shared patterns, rather than offering a deeply contextualised account of any single place. We acknowledge that this limits the ethnographic and embodied detail we are able to provide surrounding how people in these towns experience health and wellness in relation to the cycle of social violence. Future research could address these limitations by applying this framework in longitudinal studies in different geographic and cultural contexts.

This paper offers an initial theoretical proposition, the cycle of social violence, as a way to conceptualise how these distinct but interconnected forms of violence sustain health inequalities; we hope it serves as a foundation for further empirical and conceptual work that can deepen, refine, and critically engage with this framework across diverse contexts. To that end, we have identified the following avenues for future research. Qualitative researchers should explore how these forms of violence interact in different socioeconomic and political settings. For example, in the USA, where health inequalities are shaped by a privatised healthcare system (Goodair and Reeves, 2024) and entrenched racial disparities (Macias-Konstantopoulos et al., 2023), applying this framework could illuminate how structural violence operates in a context with weaker social protections and a different ideological approach to welfare. Similarly, in Europe, comparative studies could examine how varying welfare state models, such as the social-democratic systems of Scandinavia, mediate the interactions between structural, slow, and symbolic violence. Adopting an embedded, ethnographic approach would allow for contextually rich insight into how the cycle of social violence operates across distinct settings. Such research could also explore how resistance to these forms of violence emerges in different settings, identifying the social movements, policy shifts, and community actions that successfully challenge this cycle.

Future quantitative researchers should operationalise the cycle of social violence framework across spatial contexts to evaluate its explanatory power in the manifestation of health inequalities. Such research could develop composite indicators for each form of violence (eg. structural violence might be captured through metrics such as long-term unemployment, welfare spending cuts, or measures of service disinvestment). These indicators could then be used in spatial and multilevel modelling to explore how the co-occurrence and interaction of these forms of violence predict variations in health outcomes across regions. For example, researchers could test whether areas experiencing both prolonged structural and symbolic violence show worse health outcomes than areas facing structural violence alone, thereby empirically examining the interactive, compounding nature of the cycle. Longitudinal datasets could also be used to investigate whether health trajectories worsen over time in areas where these forms of violence are most acute. In doing so, quantitative research could help assess whether the patterns identified in this study are observable at scale and across contexts, serving as a complement to qualitative and ethnographic approaches.

## 5 Conclusion

Health inequalities are not accidental or inevitable, they are a form of violence, inflicted through political and economic choices that systematically harm some groups of people while protecting the interests of others. This study has demonstrated how structural, slow, and symbolic violence interact to create a self-perpetuating cycle that deepens disadvantage, erodes resistance, and obscures accountability. Addressing health inequalities in affected communities requires a direct reconciliation with whether or not we wish to live in an equitable society that allows everyone, regardless of where they are born, the same opportunities for health and well-being. The cycle of social violence highlights that health inequalities are a matter of social justice, rooted in the need to rectify fundamental inequities within society.

## Data Availability

The data that has been used is confidential.
